# Cognitive, functional and physical activity impairment in elderly with Alzheimer's disease

**DOI:** 10.1590/1980-57642018dn12-010004

**Published:** 2018

**Authors:** Renata Valle Pedroso, Danilla Icassatti Corazza, Carla Andreza de Almeida Andreatto, Thays Martins Vital da Silva, José Luiz Riani Costa, Ruth Ferreira Santos-Galduróz

**Affiliations:** 1 PhD. Institute of Biosciences, Department of Physical Education, UNESP - University State of São Paulo, Physical Activity and Aging Lab (LAFE), Rio Claro, São Paulo, Brazil; 2 PhD. Department of Physical Education, State University of Ponta Grossa - UEPG, Ponta Grossa, Paraná, Brazil; 3 Ms. Institute of Biosciences, Department of Physical Education, UNESP - University State of São Paulo, Physical Activity and Aging Lab (LAFE), Rio Claro, São Paulo, Brazil; 4 PhD. Instituto Federal Goiano (IF Goiano) - Campus Morrinhos, Goiás, Brazil; 5 PhD. Institute of Biosciences, Department of Physical Education, UNESP - University State of São Paulo, Physical Activity and Aging Lab (LAFE), Rio Claro, São Paulo, Brazil. Center of Mathematics, Computing and Cognition, University Federal of ABC - UFABC, Santo André, São Paulo, Brazil

**Keywords:** dementia, cognition, functioning, exercise, motor activity, demência, cognição, funcionalidade, exercício, atividade motora

## Abstract

Alzheimer's Disease (AD) is a progressive degenerative disorder that negatively affects quality of life of patients and family members.

**Objective:**

The aim of this study is to compare the cognition, level of physical activity and functioning of elderly individuals with mild AD and those without dementia.

**Methods:**

The study comprised 24 elderly with mild AD (mean age = 76.9 ± 5.3 years) and 30 elderly without dementia (mean age = 74.1 ± 5.6 years). The following instruments were applied to evaluate cognitive functions: MMSE; Frontal Assessment Battery; Clock Drawing Test; Corsi Blocks, and Verbal Paired Associates. Event-related potential P300 was used to evaluate cognitive processing. The Modified Baecke Questionnaire For Older Adults was applied to evaluate the level of physical activity together with use of a pedometer for 7 consecutive days. For the evaluation of the functioning, the Direct Assessment of Functional Status-Revised scale and functional tests were used.

**Results:**

There was a significant difference between the groups in level of physical activity and functioning, except on the test evaluating flexibility.

**Conclusion:**

Elderly with AD had cognitive, functional and physical activity deficits which can manifest even in the early stages of the disease.

## RESUMO

A doença de Alzheimer é um processo neurodegenerativo e progressivo que afeta, negativamente, a qualidade de vida do paciente e de seus familiares.

**Objetivo:**

Comparar a cognição, nível de atividade física capacidade funcional de idosos no estágio leve da doença de Alzheimer e de idosos sem demência.

**Métodos:**

Participaram deste estudo 24 idosos no estágio leve da doença de Alzheimer (média de idade = 76,9 ± 5.3 anos) e 30 idosos sem demência (média de idade = 74,1 ± 5,6 anos). Foram utilizados os seguintes instrumentos de avaliação cognitiva: MEEM, Bateria de Avaliação Frontal, Teste do Desenho do Relógio, Blocos de Corsi e Pares Verbais Associados. Além disso, foi realizado o exame eletroencefalográfico P300 para avaliar o tempo de processamento de informação. Para quantificar o nível de atividade física foi utilizado o Questionário Baecke Modificado para Idosos e o pedômetro, que foi utilizado por 7 dias consecutivos. A funcionalidade foi avaliada através da escala Direct Assessment of Functional Status-Revised, juntamente com testes funcionais que avaliaram os componentes da capacidade funcional.

**Resultados:**

Foram encontradas diferenças significativas entre os grupos para o nível de atividade física e funcionalidade motora, com exceção da avaliação da flexibilidade.

**Conclusão:**

Idosos com doença de Alzheimer apresentam prejuízos cognitivos, funcionais e de nível de atividade física, os quais encontram-se alterados nos estágios iniciais da doença.

The progression of Alzheimer's disease (AD) is characterized by neuronal degeneration that results in a gradual decline in numerous cognitive processes and cortical activity.^1^ In addition to these cognitive impairments, as the disease progresses AD patients can develop impairments to motor skills including reduced levels of flexibility, agility, strength, balance and aerobic resistance.^2^^-^ 5 Such impairments can directly affect the elderly's ability to perform activities of daily living (ADLs).^6^ ADLs require integrity not only of cognitive components, but also of the motor components of functional capacity (flexibility, resistance, strength, agility, balance, rhythm, motor coordination and speed) in order to be carried out effectively and safely.^6^

Therefore, the cognitive and functional deficits of AD progression are an important issue since, as they are strongly associated with ADLs, their impairment can entail reduced autonomy followed by increased risk of institutionalization; death; depression and decreased level of physical activity.^1^^,^ 4 Some studies have found that elderly with AD exhibit a low level of physical activity but have not specifically investigated individuals in the mild stage of AD.^4^^,^ 7 Given this perspective, it is important to investigate which impairments might be present in the early stages of AD. Hence, the objective of this study was to compare cognition, level of physical activity and functioning between elderly individuals in the early stage of AD with elderly individuals without dementia.

## METHODS

### Subjects

The sample comprised 30 elderly without dementia and 24 elderly with a clinical diagnosis of probable AD. Subjects were recruited from the Functional and Cognitive Kinesiotherapy Program in Older Adults with Alzheimer's Disease (PRO-CDA) and from the Program of Physical Activity for Senior Citizens (PROFIT), offered by the Department of Physical Education of the Institute of Biosciences at the Universidade Estadual Paulista "Julio de Mesquita Filho" (UNESP), Rio Claro campus.

The inclusion criteria for the elderly without dementia were: age between 65 and 90 years; no clinically relevant depressive symptoms and/or untreated symptoms; no diagnosis of AD; preserved ambulation; no hearing impairment; and no other neuropsychiatric conditions. The inclusion criteria for the elderly with AD were: diagnosis of probable AD according to the Diagnostic and Statistical Manual of Mental Disorders (DSM-IV),^8^ and elderly with AD in the mild stage of the disease according to the Clinical Dementia Rating (CDR).^9^^,^ 10

Table 1 shows the general characteristics of the group without dementia and the group with AD that participated in the study.

**Table 1:** Means and standard deviations of general characteristics and cognitive evaluation of the group without dementia and the group with AD.

	Elderly without dementia (N = 30)	Alzheimer's disease (N = 24)	P	Effect size (d)
Age (years)	74.1 ± 5.6	76.9 ± 5.3	0.06	-
Education level (years)	4.5 ± 3.7	5.0 ± 4.1	0.66	-
MMSE (points)	24.6 ± 4.0	19.8 ± 4.5	<0.01*	1.12
FAB	13.5 ± 2.9	10.5 ± 3.7	0.04*	0.38
GDS-30 (points)	6.9 ± 5.4	5.9 ± 4.6	0.54	-
Height (meters)	1.53 ± 0.05	1.57 ± 0.07	0.03*	0.16
Weight (Kg)	62.7 ± 10.3	63.9 ± 9.9	0.68	-
BMI (Kg/m^2^)	26.5 ± 4.0	25.9 ± 3.5	0.55	-

MMSE: Mini-Mental State Examination, FAB: Frontal Assessment Battery, GDS-30: Geriatric Depression Scale, Kg: Kilograms, BMI: Body Mass Index;

* p≤0.05. Student's t-test.

The elderly without dementia, and the caregivers of these elderly with AD, signed an Informed Consent Form. The Research Ethics Committee of the Universidade Estadual Paulista approved both the consent form and the research Project under protocol number 3174.

### Data collection

A psychiatrist specialized in geriatrics evaluated the participants on their first visit to the laboratory in order to confirm the diagnosis and staging of AD. The first sessions were then scheduled for evaluations of cognitive performance and level of physical activity followed by application of the functional tests.

### Evaluation instruments

#### Cognitive evaluation and depressive symptoms

The Mini-Mental State Examination was used to evaluate overall cognitive function.^11^^,^ 12 The Frontal Assessment Battery was used for the evaluation of participants' frontal functions (executive and attention).^13^^,^ 14

Cognitive processing speed was evaluated by the auditory event-related potential P300. For this purpose, the oddball paradigm was adopted using a device for the electrophysiological auditory evaluation (CONTRONIC, model "MASBE ATC Plus").

The reference electrodes were placed on the right and left mastoid, and the electrode for recording P300 was placed on the vertex, at a high mid-frontal position (position Fz), according to the 10-2 international system. We applied a sequence of binaural auditory stimuli, containing two signals of the same intensity (90 dB). Within the sequence, the standard stimulus (1000 Hz) was presented 80% of the time, while the rare stimulus (2000 Hz) was randomly interposed between standard stimuli 20% of the time. A total of 300 stimuli were emitted, each of lasting 100 ms.

The Geriatric Depression Scale (GDS-30) was applied to evaluate depressive symptoms.^15^

#### Evaluation of level of physical activity

The level of physical activity was assessed by the Modified Baecke Questionnaire for Older Adults (MBQ)^16^ and a Yamax Digiwalker pedometer for seven consecutive days.

#### Evaluation of functioning

Functioning was assessed using the Direct Assessment of Functional Status (DAFS-R) instrument.^17^ Several functional tests were applied: 6-minute Walk Test^18^ to assess aerobic resistance, 30-s Chair-Stand Test^17^ and Arm Curl Test (AAHPERD)^19^ to assess strength resistance of the lower and upper limbs, respectively, Berg Functional Balance Scale (BFBS)^20^ to evaluate static and dynamic balance and balance recovery, the Timed Up-and-Go test (TUG)^21^ to evaluate basic functional mobility, and finally the Wells Sit-and-Reach Test^22^ to evaluate flexibility.

### Statistical analysis

The Shapiro Wilk test was initially used to analyze data distribution. Student's *t-* test was used for data displaying a normal distribution. For the groups that had a non-normal distribution, the Mann Whitney's U test was employed. The level of significance was set at 5% for all analyses. All data were expressed descriptively. The statistical software Statistica version 7.0 was used for all data analyses.

## RESULTS

### Cognitive functions

**Figure 1:**
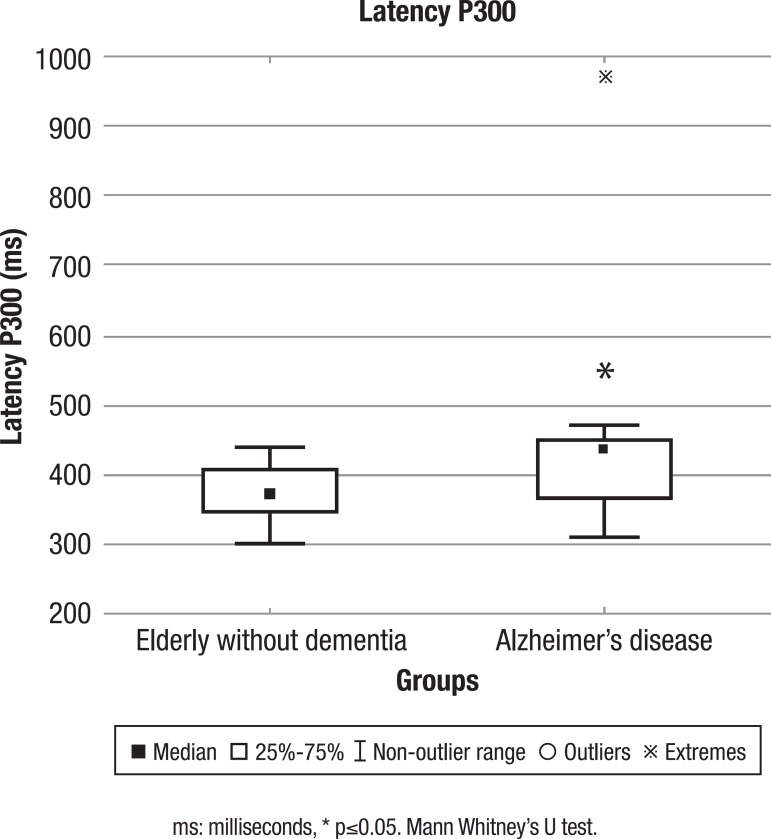
Median and interquartile range of P300 (ms) latency of the group without dementia and the group with AD.

The elderly with mild AD exhibited impairment of cognitive and frontal functions when compared with the individuals without dementia (Table 1). Additionally, the P300 evaluation showed that the group with AD had longer latency, indicating slower cognitive processing than the group without dementia (p = 0.01) (Figure 1).

### Level of physical activity

According to both evaluation instruments, the elderly with mild AD had lower levels of physical activity (Table 2). Moreover, the analysis of the MBQ showed that the groups differed mainly for performance of household chores (Table 2).

**Table 2:** Means and standard deviations/median and interquartile range of functional tests and level of physical activity of the group without dementia and the group with AD.

	Elderly without dementia (N = 30)			Alzheimer's disease (N = 24)		P	Effect size (d)
	Mean ± standard deviations	Median/Interquartile range		Mean ± standard deviations	Median/Interquartile range		
30-s Chair-Stand Test	-	14.5/12.0-19.0		-	11.0/9.0-13.5	<0.01*	1.10
Arm Curl Test	-	21.0/18.0-29.0		-	17.0/13.0-21.0	<0.01*	1.74
Wells Test	-	22.0/18.0-28.0		-	20.0/13.0-25.5	0.17	-
TUG Time	7.3 ± 1.8	-		8.6 ± 1.9	-	<0.01*	0.70
TUG Steps	-	54.0/52.0-56.0		-	16.0/14.0-88.0	<0.27	-
BFBS (points)	-	54.0/52.0-56.0		-	52.0/50.0-54.0	0.04*	0.43
MBQ Household Chores	-	2.0/1.6-2.4		-	1.2/0.8-1.8	<0.01*	1.37
MBQ Sports Activities	-	0.0/0.0-2.2		-	0.0/0.0-0.0	0.07	-
MBQ Leisure	-	0.5/0.0-1.2		-	0.0/0.0-1.4	0.41	-
MBQ Total Score	-	3.0/2.4-5.5		-	1.9/1.0-4.3	<0.01*	0.72
Pedometer (steps)	-	6154/4317-8556		-	1509/605-4411	<0.01*	0.93

TUG: Timed Up-and-Go; BFBS: Berg Functional Balance Scale; MBQ: Modified Baecke Questionnaire for Older Adults

* p ≤ 0.05. Student's t-test for TUG time and Mann Whitney's U test for the other items.

### Functional tests

The elderly with AD had higher deterioration of strength resistance of lower and upper limbs and worse balance, agility/mobility and aerobic resistance than the elderly without dementia. No significant differences were detected between the groups for flexibility (Table 2).

### Functioning

The elderly with AD showed greater deterioration of functioning when compared with the elderly without dementia (Table 3). The groups differed on the sub-items *time orientation, communication, finance, shopping and grooming.* There were no differences for the sub-item *eating* (Table 3).

**Table 3:** Means and standard deviations/median and interquartile range on the Direct Assessment of Functional Status (DAFS) of the group without dementia and the group with AD.

	Elderly without dementia (N = 30)			Alzheimer's disease (N = 24)		P	Effect size (d)
	Mean ± standard deviations	Median/Interquartile range		Mean ± standard deviations	Median/Interquartile range		
DAFS Time Orientation	-	16.0/14.0-16.0		-	12.0/9.0-14.0	<0.01*	1.35
DAFS Communication	-	14.0/12.0-15.0		-	10.0/7.0-13.0	<0.01*	0.94
DAFS Finance	20.5 ± 8.2	-		13.6 ± 5.9	-	0.01*	0.96
DAFS Shopping	-	14.0/13.0-17.0		-	8.0/2.0-15.0	<0.01*	1.20
DAFS Grooming	-	13.0/13.0-13.0		-	11.5/10.0-13.0	<0.01*	1.41
DAFS Eating	-	10.0/10.0-10.0		-	10.0/10.0-10.0	0.60	-------
DAFS Total Score	-	85.5/73.0-97.0		-	62.5/55.0-72.0	<0.01*	1.53

DAFS: Direct Assessment of Functional Status.

* p≤0.05. Student's t- test for DAFS finance and Mann Whitney's U test for the other items.

## DISCUSSION

### Cognitive functions

The interpretation of the data found in this study revealed that the elderly with AD in the mild stage of the disease had greater deterioration in global cognitive functions and also frontal functions when compared with the elderly without dementia, where these results agree with other studies.^23^

The latency of P300 was analyzed in both groups using an electroencephalographic exam to identify possible differences in cognitive processing speed. The elderly with AD had longer P300 latency (416 ms) than the elderly without dementia (375 ms). In their review study, Pedroso et al.^24^ analyzed several studies that also made this comparison. However, few of them evaluated only elderly in the early stage of AD. Pedroso et al.^24^ found P300 values in the 358 ms-458 ms range. The results of the present study were also within this range, showing cognitive processing delay among the elderly in the mild stage of the disease.

### Level of physical activity

The level of physical activity of the elderly with mild AD was lower than that of the elderly without dementia, both according to the MBQ and the pedometer, findings that corroborate results reported in the literature.^4^^,^ 7 ^,^ 25

Lima et al.^7^ used a pedometer to evaluate the level of physical activity of patients with AD, and found low levels (4400 steps). Moreover, the authors suggested that 6000-6500 steps a day would be the recommended number for this specific group. The elderly with AD performed a median of only 1509 steps/day. The elderly without dementia had a median of 6154 steps/day, within the expected range according to Tudor-Locke et al.^26^

The MBQ results also showed that the elderly with AD had a low level of physical activity (median of 1.9 points on the test). This result is in line with those of other studies in the literature that detected a mean of 2.59 points^4^ and 1.8 points.^27^ None of these studies, however, evaluated the level of physical activity of elderly individuals specifically in the mild stage of AD.

The analysis of MBQ domains revealed that the elderly with AD had lower performance for household chores in the very early stages of AD. This reduction may be associated with the cognitive impairment resulting from the condition.

### Functional tests

Elderly with mild AD showed lower strength resistance of lower limbs. Eggermont et al.^5^ observed no differences between groups, whereas Manckoundia et al.^2^ found that the elderly with AD in the mild and moderate stages of the disease had poorer performance on the sit-to-stand and back-to-sit test.

The elderly with AD also showed impaired resistance of the upper limbs. The finding of altered lower and upper limb resistance is fundamental, since this muscle function is highly recruited in the performance of daily living activities^28^^,^ 29 and therefore directly affects the autonomy of the individual with dementia.

The only component of functional capacity that did not show significant difference between groups was flexibility. We found no studies in the literature comparing flexibility between elderly with and without AD. One of the explanations could be related to the test, which may not have been sensitive to detect few differences between groups. Another reason might be that flexibility suffers little influence from the cognitive declines of AD.

The elderly in the early stage of AD had impaired functional balance, with a median of 52 points on the BFBS versus 54 points in the elderly without AD. Pedroso et al.^30^ detected a mean of 49 points on the BFBS. The participants of the study, however, were in the early and mild stages of the disease. Kato-Narita et al.^3^ found greater balance deterioration (also evaluated by the BFBS) in elderly in the moderate stage of AD compared with elderly without AD. Our study found evidence that this deterioration might already be present in the early stage of AD.

According to the evaluation of agility and mobility by the TUG test, elderly with AD performed the test in 8.6 seconds. Coelho et al.,^31^ in a recent study comparing elderly with AD in several stages of the disease, found that those in the mild stage performed the test in 9.2 seconds. Some studies have shown that elderly with AD have greater mobility/agility impairment than those without dementia.^4^^,^ 5

Rikli and Jones^28^ reported that elderly with AD are already within the range of impairment of functional independence, since 8.5 seconds on the test is the cutoff point identified as a predictor of falls among the older elderly, which occur at a rate of 4 to 5 falls per year.^32^

Finally, the last component of the functional capacity evaluated was aerobic resistance. The group with AD had poorer performance (406 meters) than the group without dementia (443 meters) on the 6-minute Walk Test. This result corroborates data in the literature, according to which elderly with dementia have reduced cardiorespiratory capacity.^33^ Rikli and Jones^28^ suggested a cut-off point on the 6-minute Walk Test for elderly aged over 85 years of 400 to 460 meters in order to be considered independent.

### Functioning

The elderly with AD had a median score of 62.5, lower than that of the group without dementia (85.5 points), suggesting impaired functioning and decreased ADLs in the elderly with AD. Zanetti et al.^34^ also found differences between elderly with and without AD. However, the authors evaluated elderly at different stages of the disease in the same sample.

Several studies have suggested that this decrease in ADLs is associated not only with cognitive decline, but also with functional decline and behavioral disorders present during the progression of AD. 35

The AD group in the present study showed impairment for *time orientation, communication, finance, shopping and grooming.* The only variable evaluated that did not differ between the groups was *eating* .

The final score of the elderly in the mild stage of AD in the cited study was similar to the score found in the present study (58 points). The authors, however, did not investigate for which activities the elderly were impairment. Consequently, we can infer that *eating* may have been preserved in the early stages of AD. In fact, the first ADL altered in elderly with dementia is *time orientation,* followed by more complex tasks that require preserved cognitive functions, such as *finance* and *shopping* .^16^ The evaluation of functioning by the DAFS is important to assess the elderly with or without dementia for performance of ADLs.

The authors of the present study acknowledge several limitations that should be considered when interpreting the data. One of these limitations is related to the small sample size and the lack of sample size estimation. In addition, the assessment of the level of physical activity had some limitations. For instance, pedometers are unable to measure the intensity of physical activity. However, the questionnaires applied are subjective and the caregiver answers the questions about the patient. Based on the interpretation of the results obtained in the present study, it can concluded that the elderly with AD exhibited cognitive and functional impairment, and reduced physical activity, which can be present even in the early stages of the disease. The relevance of the present study is that it can help support the creation of intervention programs targeting the population with Alzheimer's disease, since these elderly individuals have specific needs, as highlighted by this study.
